# Impact of Family Involvement in Cardiac Rehabilitation—Insights from a Literature Review

**DOI:** 10.3390/jcm14186468

**Published:** 2025-09-13

**Authors:** Gabriela Popescu, Alexandra Maștaleru, Andra Oancea, Alexandru-Dan Costache, Cristina Andreea Adam, Carmen Rîpă, Carmen Marinela Cumpăt, Maria Magdalena Leon

**Affiliations:** 1 Clinical Rehabilitation Hospital, 700661 Iasi, Romania; gabriela.popescu@umfiasi.ro (G.P.); andra.oancea@umfiasi.ro (A.O.); dan-alexandru.costache@umfiasi.ro (A.-D.C.); cristina-andreea_adam@umfiasi.ro (C.A.A.); marinela.cumpat@umfiasi.ro (C.M.C.); maria.leon@umfiasi.ro (M.M.L.); 2 Department of Medical Specialties I, “Grigore T. Popa” University of Medicine and Pharmacy, 700115 Iasi, Romania; 3 Department of Microbiology, Faculty of Medicine, “Grigore T. Popa” University of Medicine and Pharmacy, Universitatii Street No. 16, 700115 Iasi, Romania; ripa.carmen@umfiasi.ro; 4 Department of Preventive Medicine and Interdisciplinarity, “Grigore T. Popa” University of Medicine and Pharmacy, Universitatii Street No. 16, 700115 Iasi, Romania; 5 Department of Medical Specialties III, “Grigore T. Popa” University of Medicine and Pharmacy, 700115 Iasi, Romania

**Keywords:** rehabilitation, cardiovascular disease, family, social, support

## Abstract

Conventional medicine relies solely on the interaction between the doctor and the patient, not necessarily including the family in discussions about therapy and diagnosis. Nevertheless, recent research indicates that the presence of a family member during a doctor’s visit improves both communication between the doctor and the patient, as well as the patient’s understanding of information related to their health condition. Additionally, by adopting this approach, family members can better understand what obligations arise at this stage and the patient’s needs. Studies have shown that collaboration with family members has significantly reduced mortality rate and improved their quality of life, as the family strengthened their confidence in their abilities by providing moral and emotional support. Even though family support and involvement seem to be very important, sometimes patients have reported that overly active family involvement becomes intrusive, as it violates their privacy and disrupts their autonomy. That is why it is important for family members directly involved in recovery to learn to respect the boundaries set by the patient. Both the patient and their family experience stressful moments during this recovery period, which necessitate their attention. The involvement of family members in the recovery process reduces their levels of anxiety and stress, as they can closely monitor the patient’s progress. Additionally, integrating family members into the rehabilitation program can provide further benefits for the patient, as the presence of a family member leads to increased comfort and motivation.

## 1. Introduction

Following hemodynamic stabilization, patients undergoing coronary artery bypass graft surgery, myocardial infarction, or heart transplant participate in a cardiovascular rehabilitation program. This program will help the patient return to their pre-surgery life by managing risk factors, implementing targeted physical exercises, and providing psychological support [1,2]. The recovery process for patients with cardiovascular pathology can be divided into 3 phases. These are individualized according to the type of surgery and associated pathologies. During the first few days following surgery, the patient undergoes an acute recovery process that often includes light walking, exercises performed in bed or by the bedside, psychotherapy, nutritional counseling, and educational sessions on improving their lifestyle and reintegrating into society. The following two phases involve more advanced weight-bearing, treadmill, or bicycle activities, as well as physiotherapy. The healing phase lasts about six months, and the post-healing phase follows after six months and lasts for the rest of the patient’s life [3,4]. In addition to training to increase physical endurance, muscle strength, stability, and coordination, patients are instructed to give up unhealthy habits such as smoking and alcohol consumption, to reach an ideal weight, and to keep associated pathologies under observation. Furthermore, psychological intervention is crucial for cardiac patients because it improves long-term results by lowering stress, anxiety, and depression—all of which are risk factors for cardiovascular disease—and enhances adherence to the rehabilitation program [5,6]. Ambrosetti and his colleagues mention the importance of family integration in the cardiovascular rehabilitation programme and highlight the importance of psychological support from the family, but without specifying the influence on physical health or adherence to the rehabilitation programme [7].

Inevitably, the psychological well-being of patients is profoundly impacted following major surgery. Consequently, this affects the process of recuperation. Evidently, a significant number of patients encounter moments of worry upon commencing the rehabilitation program, as they harbor apprehension regarding potential difficulties during physical activity or possibly a reoccurrence of a cardiac event. Linked to this apprehension, depression arises, and the patient experiences poor appetite, fatigue, and lack of motivation [8]. Despite the positive impact of cardiovascular rehabilitation programs on mental well-being, not all patients attain a condition of balanced mental health [9]. For instance, individuals participating in a study carried out in Sydney, Australia, had an evaluation prior to being enrolled in a cardiovascular rehabilitation program. Out of 4784 patients, 18% received a diagnosis of moderate to severe depression, 28% experienced high levels of anxiety, and 13% manifested symptoms related to moderate-severe stress. By the conclusion of the program, a mere 9% of individuals afflicted with depression reported any improvement, while among those struggling with anxiety and stress, just 15% and 8%, respectively, achieved any improvement. This group of patients was more prone to discontinue their participation in the rehabilitation program. Heightened levels of anxiety and stress result in depression, while depression and stress in turn bring on anxiety [10].

These accumulating conditions compel the patient to withdraw from society, therefore heightening the probability of a cardiovascular incident recurring [8]. Family support is crucial in such circumstances and can drastically impact engagement in the rehabilitation program. Within this timeframe, patients often require assistance with daily domestic tasks, such as cooking, shopping, and cleaning. The family is the nearest and most suitable source to address these issues, therefore ensuring the patient’s sense of security. Furthermore, family members can provide sincere moral encouragement both in the early stages of recuperation after surgery and during the later stages of rehabilitation. Moreover, the family can facilitate the efforts of reintegrating into society and achieving psychological rehabilitation [11,12].

Medical guidelines emphasise the importance of family involvement in cardiovascular rehabilitation programmes, but none of them clearly highlight the short- and long-term effects it can have. Therefore, this review aims to highlight both the positive and negative impacts of family involvement in the recovery process.

## 2. Materials and Methods

**Figure 1:**
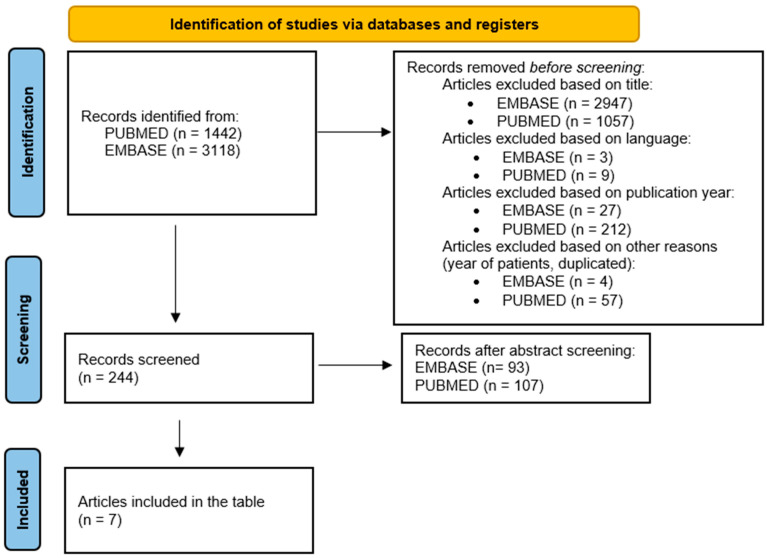
The selection process of the articles highlighting the importance of family involvement in cardiovascular rehabilitation.

The purpose of this review is to evaluate the impact of family in the cardiovascular recovery process. The inclusion criteria for this review included studies that addressed the importance of family support in the cardiovascular recovery process, even if this was not the central theme of those articles. Articles that were not written in English and those that included participants under 18 years of age were excluded. We excluded review papers, abstracts, editorials, statements, comments, and conference papers. As search terms, we used the association between the following words: “family”/”relatives”/”social” and “support”, “cardiovascular rehabilitation”. These studies were selected based on their relevance to the review theme, focusing on studies with the theme we addressed as their main topic (Figure 1).

## 3. Results

Although a broader body of literature addresses family involvement in cardiac rehabilitation, for the purpose of this review, we selected seven studies that we considered the most relevant and illustrative (Table 1). These articles synthesize the core evidence and perspectives on this subject, without claiming to represent an exhaustive list of all available publications.

## 4. Discussion

### 4.1. Does Family Support Matter in the Cardiovascular Recovery Process?

Conventional medicine relies exclusively on the doctor-patient interaction, without necessarily including the family in discussions regarding therapy and diagnostics. Nevertheless, recent research indicates that the presence of a family member during a doctor’s visit enhances both good communication between the doctor and patient and improves comprehension of health-related information. Furthermore, by adopting this approach, family members can gain a deeper understanding of their obligations during this phase and the patient’s requirements. This is particularly important, as several instances involve a lack of awareness regarding the gravity of the medical condition, leading family members to expect the patient to resume previous activities and habits promptly after the surgery [19,20]. Active family involvement is crucial for the patient. For instance, a significant number of individuals require someone to oversee the administration of medication, ensuring strict adherence to the allocated dosage and schedule. They feel let down when their children or close friends fail to demonstrate concern for their well-being [16]. Even so, it is crucial for the family to demonstrate understanding and avoid causing extra stress for the patient by consistently reminding them of the rules they must conform to [19,20].

Concerning family collaboration, males often have trouble expressing the adverse emotions that directly affect their lives after surgery. Conversely, women are more keenly impacted by the domestic chores they must manage. Unfortunately, it appears that in certain instances, women prioritize their household obligations above their involvement in the rehabilitation program and fail to provide time for hospital visits. Furthermore, research indicates that women who are currently employed or responsible for a spousal partner do not receive support from their families to engage in the rehabilitation program. Furthermore, for women belonging to ethnic minorities, collaborating with a man during the process of rehabilitation is regarded as religiously unacceptable [21,22].

Published in 2015, a study investigates whether a hybrid cardiac rehabilitation program, including family members, can enhance patients’ quality of life by reducing the perceived stress and anxiety linked to a myocardial infarction in comparison to a conventional cardiac rehabilitation program. The study included a total of seventy patients with an average age of 61.40 ± 12.83 years, of which 35 were assigned to the intervention group and 35 to the control group. Out of the total, 65.7% were male, 98.6% were married, and they belonged to a family of around 5.37 ± 1.91 members. The caregiver’s responsibilities included participating in all educational sessions with the patient, providing updates on the patient’s home progress, and supervising the patient’s activities during therapy sessions. The beneficence of this form of recovery was evaluated using six questionnaires that measured quality of life, health status, and depressive and anxiety status. In conclusion, the study revealed that patients who engaged in the rehabilitation program with their family members experienced more significant enhancements in their quality of life, physical well-being, and mental health compared to those who participated in the conventional cardiovascular rehabilitation program. Although there were no significant differences between the two groups, it seems that the support and optimistic atmosphere from the family resulted in both physical and physiological advantages [23].

In 2021, a study conducted interviews with 14 cardiovascular recovery practitioners to gather their perspectives on family involvement in the process. They stated that the family’s presence at the doctor’s appointment gave the patients peace of mind because there was another person with them who understood what the recovery process entailed. Very often, patients would come to the doctor demotivated and unwilling to engage in conversation, and in these cases, the family was a key intermediary. Moreover, in some cases, family members were actively involved in the recovery, trying to do the exercises and follow the rules, thus becoming a role model for the patient [15].

For a double-blind randomized trial at Shariati Hospital, 70 patients who experienced myocardial infarction were included, together with a small number of family members chosen by the patients. The purpose of the study was to compare the effectiveness of cardiovascular recovery with family members versus the effectiveness of conventional cardiovascular recovery. The follow-up period spanned a decade, from 2012 to 2023. The study participants selected individuals who were then allocated to participate in recovery stages 3 and 4, with phase 2 being entirely voluntary. Each patient and caregiver was provided with identical information regarding the exercises to be performed. Every group of patients adhered to a 2 h daily exercise regimen. Throughout the exercise sessions, family members assumed the responsibility of overseeing and providing guidance to the patient. Patient supervision in the control group was only carried out by the family, without any additional involvement. The study concluded that patients collaborating with family members significantly reduced their mortality rate, while the control group experienced a mortality rate four times higher. Furthermore, the patients expressed a favorable improvement in their quality of life, as the family bolstered their self-assurance in their own capabilities by providing moral and emotional assistance [13].

During an interview conducted by Natalie A. Hagan et al., patients expressed that the involvement of their family during the recovery phase influenced their decision-making process regarding lifestyle modifications and engagement in recovery program services. Although the family did not play a significant role in determining the outcome for some individuals, the provision of basic emotional support had a profound impact throughout this challenging time [18]. Additionally, a retrospective study published in 2024 highlights that a cardiovascular rehabilitation program based on collaboration between healthcare professionals and family improves the clinical outcomes of the rehabilitation program. Statistically significant results were observed, not only when evaluating patients’ quality of life, but also in terms of echocardiographic parameters and laboratory tests [14]. In a cross-sectional study, six weeks after hospital discharge, 169 patients with coronary artery disease answered a questionnaire about their family’s role in their cardiovascular recovery. Patients were asked whether their family helped or hindered them in the recovery process. Researchers wanted to know if the patient’s family members helped with household chores, provided physical and moral support during the recovery process, and if they were interested in learning as much as possible about the patient’s condition. Additionally, by using the questionnaire to analyze the harmful effects of such a serious illness on a family, the researchers aimed to determine whether the family experienced anxiety or depression during this period and whether their relationships deteriorated. Ultimately, the researchers concluded that most patients found genuine support in their families, and their relatives’ positive and optimistic attitudes motivated them. Regrettably, this period has adversely impacted families psychologically and financially in certain instances, a situation that some patients have voiced concerns about [17].

When it comes to cardiovascular recovery, diet plays a crucial role, as an inadequate diet can lead to an increase in cardiovascular risk factors. Patients who have experienced a major cardiac event should limit their salt intake, stop consuming alcohol and ultra-processed foods, and incorporate more fruits, vegetables, whole grains, and fish into their daily diet [24]. It appears that the process of adopting a new diet varies depending on the patient’s gender and their role within the family. In South Asia, the adoption of a new diet by a man often encourages the entire family to adapt to the new habits. On the other hand, women do not have the same advantage. In most cases, they cook for themselves and their family, and the pain and fatigue after surgery make them give up on a new diet and eat like everyone else. However, in some cases, the children take over meal preparation, easing the burden on the sick person [12].

Fortunately, in many cases, wives want to adopt the new lifestyle together with their partners. They prefer to take full responsibility for meal preparation and adhering to new habits, which motivates them and makes their partners much more optimistic when it comes to the recovery process [25].

### 4.2. Does Family Involvement Negatively Impact Cardiovascular Recovery?

Even though family support and involvement seem to be very important, sometimes patients have reported that overly active family involvement becomes intrusive, as it violates their privacy and disrupts their autonomy [15]. The patients express a strong desire to regain their independence promptly, which contradicts the families’ inclination to provide them with maximum protection during the initial months after surgery, leading to conflicts between them [12]. Additionally, especially after the patient recovers from a myocardial infarction, the family tends to be very protective, affecting the patient’s independence [15]. During an interview in a study, a patient reported that to protect him, his family started restricting him from leaving the house, intentionally creating distance between him and his friends. This social isolation, stemming from the desire to avoid another cardiac event, can emotionally affect the patient, leading to the opposite of the intended outcome [16].

Most patients with cardiovascular issues have unhealthy habits, such as alcohol consumption or smoking. After being discharged from the hospital, they receive a recommendation to give up these vices as they are among the main cardiovascular risk factors. Most of the time, the family puts pressure on the patient to give up these habits as quickly as possible, making them feel cornered and misunderstood. Even though giving up these two harmful vices is essential for cardiovascular recovery, the family should gently get involved in this process, as the patient will experience additional stress [26]. Furthermore, if the members of the patient’s family who are directly involved in the rehabilitation process are not emotionally stable, they have the potential to negatively impact the patient’s mood [27].

**Figure 2:**
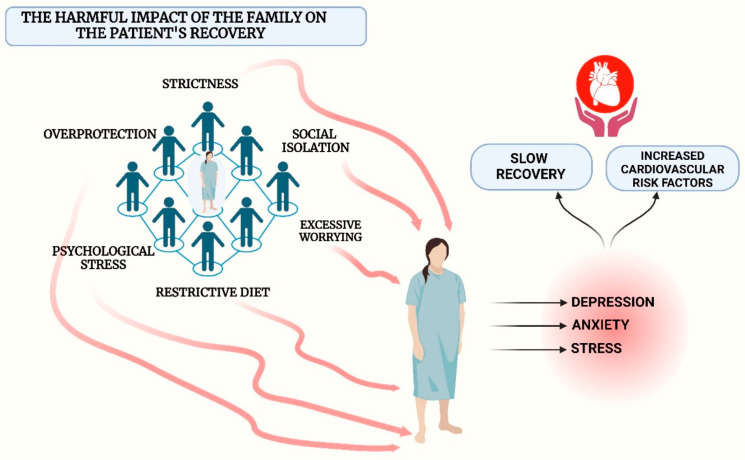
The harmful impact of the family on the patient’s recovery process.

Fortunately, in many countries, there are national programs that help patients free of charge in their battle against addictions. These global and national programs to stop smoking and alcohol consumption provide free and accessible resources to support citizens in adopting a healthy lifestyle. In the United States, “Quitline” and the National Institute on Alcohol Abuse and Alcoholism provide counseling and treatment for quitting smoking and alcohol dependence. The United Kingdom and Australia offer similar programs, such as “NHS Smokefree” and “Quitline Australia,” which include phone support, online resources, and treatment. In Romania, the Ministry of Health coordinates national programs for the prevention of smoking and alcohol dependence, providing counseling, medication treatment, and awareness campaigns. These initiatives reduce family pressure during the process of overcoming the addictions that patients had before the cardiac event [28,29,30,31,32,33]. In addition, over 70 countries have recently implemented restrictions on smoking in enclosed public spaces. Moreover, starting in 2023, at least 133 countries have raised tobacco taxes to reduce smoking rates and, implicitly, the increased number of illnesses and risk factors associated with smoking [34,35]. How family can negatively impact the cardiovascular recovery process can be seen in Figure 2.

### 4.3. Does Involvement in Cardiovascular Rehabilitation Affect the Family?

During this recovery period, not only the patient but also their family go through stressful moments. The involvement of family members in the recovery process reduces their levels of anxiety and stress, as they can closely monitor the patient’s progress. If the family is not well-informed about the patient’s needs, instead of providing a positive contribution to their life, they can become an additional source of stress [15]. Following a significant cardiovascular event, the patient is unable to resume their prior lifestyle until they have completely recuperated. Consequently, the family experiences both the financial consequences and the responsibilities that often burden their spouses or children [17,18].

When it comes to changing their lifestyle, patients try to compel their close family members to follow the same way of life. In a study, a patient’s husband recounts that most of the time he opts for a healthy diet just like her, but when he makes different choices from his wife, she criticizes him [25].

In general, when it comes to a rehabilitation program, the attention is entirely on the patient in question. Serious illnesses, such as cardiovascular disease, affect both the sufferer and their family. Patients’ life partners, who are always by their side, experience the most stress. Family members’ calmness improves the patient’s mental state and accelerates recovery, as they are with them every day. When the patient experiences a catastrophic cardiovascular episode, undergoes surgery, and needs assistance, it impacts the family [27,36,37]. It appears that younger spouses are far more likely to have symptoms such as lack of appetite, sadness, and headaches than older wives. To observe the psychological and physical effects that occurred after their husbands went through a significant cardiac event, 213 spouses of patients who had experienced a myocardial infarction were recruited for the study. Following the completion of the psychological examination, it was determined that sixty-six percent of the participants met the criteria for increased psychological stress. Sleeplessness, a persistent feeling of tension, and emotional instability were among the most prominent symptoms experienced. The most significant stress factor (75%) was the thought that the treatment and rehabilitation program would not work, followed by the patient’s emotional instability (66.7%). Younger age, the presence of minor children in the family, and a decrease in couple intimacy were the main factors that positively correlated with a higher level of stress among wives [27].

### 4.4. Socio-Economic Factors That Impact Cardiac Rehabilitation

Cardiovascular rehabilitation programs can provide cost savings of €30,500 per patient in the first year, mostly attributed to a resumption of employment, and up to €14,500 per year in the following years [38].

Despite the medical team’s recommendations, statistics from the USA, UK, and Australia show that less than 50% of patients who have experienced a significant cardiac event follow a rehabilitation program after discharge. Analyzed data revealed a correlation between involvement in outpatient rehabilitation programs and advanced age, female gender, education level, socio-economic situation, the existence of comorbidities, and lifestyle [18,38].

The financial component is a prevailing concern among both the older and younger generations. Regrettably, as a result of increased expenses, several patients frequently opt not only to withdraw from the rehabilitation program but also to abandon the whole treatment plan, thereby removing drugs they deem insignificant [39]. Numerous studies have shown that the distance between the place of residence and the hospital might provide a significant challenge, particularly for persons living in rural areas or without companionship. Many patients prefer not to impose transportation concerns on their family members. As a result, in addition to medical service expenses, many patients face the challenge of transportation costs [13,18,38,39].

A crucial determinant in the choice to engage in rehabilitation programs within the financial domain is the level of job security. The majority of patients who are company owners consented to engage in a comprehensive rehabilitation program. Conversely, employed individuals attribute greater significance to returning to the workplace. The lack of comprehension among many companies about an extended medical absence causes anxiety among patients since financial security is a crucial factor for both themselves and their families. During the interviews, some patients expressed their intention to participate in a weekend-based rehabilitation program due to this issue. Despite all this, in the case of unemployed individuals, no increase in the participation rate has been observed [18].

The culture and environment of origin may also influence participation in cardiac rehabilitation programs. Individuals hailing from rural areas or belonging to ethnic minority groups often decline involvement in the rehabilitation program. Individuals frequently express a desire to participate in such initiatives within their local community, or they may outright decline based on their community’s cultural values. Furthermore, a positive correlation exists between a limited degree of education and a low percentage of participation in these programs [39].

A study that investigated South Asian patients involved in cardiovascular rehabilitation programmes highlighted that family responsibilities usually depend on women. This situation leads to low adherence to the program because women choose to prioritise their duties as wives and mothers over their own health. The sociocultural environment they come from does not offer them support to participate in such programs; they believe that fulfilling family roles is more important than personal recovery. Additionally, several studies show that in Asian communities, time spent on recovery is perceived as a sacrifice made at the expense of the family. At the same time, Asian patients tend to attend consultations more often accompanied by children, which, combined with the language barrier, further complicates the recovery process [40]. On the other hand, patients of European origin more frequently benefit from the active support of their life partners, which is reflected in increased adherence to lifestyle changes. Thus, while Asian patients face difficulties in adopting a healthy diet, the families of European patients show a greater willingness to change their own lifestyles for the patient’s well-being. Religion is also a determining factor, especially in the case of patients from South Asia, India, or African American communities, where religious and cultural norms can limit participation in certain recovery activities. For these patients, home-based cardiovascular rehabilitation programmes could be more helpful because they allow for easier overcoming of linguistic, cultural, and religious barriers [41]. In a different context, a study conducted in Brazil highlighted that the main reason for non-participation in cardiovascular rehabilitation programmes was related to family responsibilities, with patients not receiving family approval to take the time needed for recovery [42]. The majority of the time, patients are uninterested in cardiovascular rehabilitation because they believe that physical activity would further disrupt their health. Additionally, some patients believe that they get sufficient exercise while they take care of domestic duties [16]. When compared to the lifestyle they had before the event, a significant number of patients do not feel sufficiently motivated to embrace a new way of life [39]. Some studies have found that interacting with other patients who have experienced similar issues can motivate patients to adhere to the rehabilitation program. Moreover, group-based programs for patients and their interactions seem to help significantly anxious and depressed patients psychologically, as they feel much more understood when discussing their issues with someone who has gone through the same experiences as they have. In a study published in 2021, patients reported that team recovery motivated them to follow the program and made them much more positive about their long-term progress [43]. The high number of comorbidities associated with senior age makes participation difficult, even though retired patients have fewer duties and more spare time [38,43,44]. Furthermore, if the family does not prioritize the patient’s recovery, they are less likely to be motivated to adhere [15]. Many patients seem to hesitate in making an immediate decision after surgery; instead, they prefer to return home to their families, consult with them, and only then make a decision regarding this matter [18].

When it comes to sex and gender, multiple studies have found that women have a significantly lesser presence than men. Frequently, medical personnel neglect the needs or the cultural norms of the community from which patients originate. It appears that strict weight and progress tracking have a substantial psychological influence on participants, causing them to decline involvement in group recovery programs [21,45]. Rehabilitation programs should be adapted not only to the patient’s pathology but also to the participants’ sex and gender since each has unique demands and requirements [21].

Furthermore, medical education is critical to the patient’s engagement in the rehabilitation program. Doctors should not only focus on prescription medicine or recommending exercises for patients, but also actively engage in teaching, supporting, and instructing patients and their families about cardiovascular rehabilitation [16]. Regrettably, a significant number of patients choose not to engage in such programs due to the individuals involved. Patient feedback indicated that certain clinicians they interacted with lacked empathy and displayed excessive criticism. Additionally, the lack of a systematic arrangement to address the needs of all participants led them to abandon their efforts [21,46].

**Figure 3:**
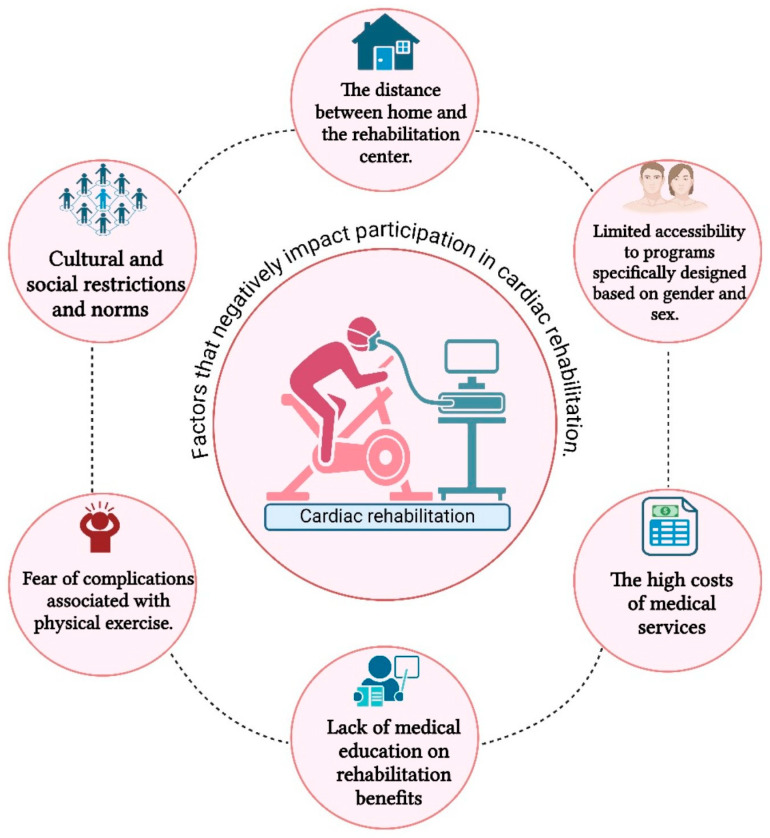
Factors that negatively impact participation in cardiac rehabilitation.

Hybrid programs that allow patients to participate from home, along with their families, are among the most feasible options today [13]. Patients who are included in family-involved cardiovascular rehabilitation programmes achieve good results in terms of symptom improvement, exercise tolerance, and quality of life. Thus, family support becomes essential for maintaining treatment adherence, monitoring symptoms, and continuing participation in programmes, thereby extending the impact of the recommendations in good practice guidelines [47]. Telemedicine is a more viable alternative, accessible even to patients residing at a considerable distance or having financial or cultural constraints that prevent them from accessing rehabilitation facilities [39]. The utilization of telerehabilitation enables the efficient monitoring of patients’ physical training, particularly those diagnosed with cardiovascular illnesses, thereby facilitating sustained adherence to therapy without the necessity of hospital visits and the potential exposure to additional risks, including other infections. Based on its demonstrated safety, efficacy, and broad acceptance among patients with cardiovascular diseases, this strategy has great potential as a tool in the continuous battle against cardiovascular disease [48]. A summary of the factors that negatively influence participation in cardiac rehabilitation programmes can be seen in Figure 3.

## 5. Conclusions

It is essential that family members, not just the multidisciplinary team, are integrated into cardiovascular rehabilitation programs, as family involvement helps patients acquire new knowledge and skills much more easily. Additionally, family can be a real help in reducing the patient’s isolation. Family participation in stress management workshops or nutritional counselling, alongside the patient, promotes a unified approach to health. Thus, cardiac rehabilitation becomes a holistic process that not only provides professional guidance but also promotes active patient involvement and family support, improving engagement, well-being, and long-term outcomes [49].

Cardiovascular rehabilitation is a very important stage in the life of a person who has undergone a major cardiac event or is diagnosed with a severe cardiovascular disease. When a patient is enrolled in a recovery program, the physician should consider not only the pathology and the steps to be taken to improve the patient’s health but also psycho-emotional and socioeconomic factors. Integrating family members into the rehabilitation program can provide several advantages for the patient since the presence of a family member leads to increased comfort and motivation.

Family members could be more sensitive to the cultural and social norms of the patient than healthcare professionals, resulting in a more comfortable setting for the patient. Furthermore, it would be more economically feasible to facilitate recovery at home, using telerehabilitation, but with the assistance of a family member. Family members directly involved in the patient’s recovery must possess emotional stability, understand and respect the patient’s privacy, and avoid imposing rigid constraints that could lead to long-term anxiety, depression, and social isolation.

A promising direction for optimizing cardiac rehabilitation programs would be the systematic integration of family members, not only through participation alongside the patient in certain stages of rehabilitation, but also through the development of structured educational programs specifically designed for families. These programs could serve as interactive learning spaces in which family members receive clear and tailored information regarding the objectives, stages, and benefits of cardiac rehabilitation, as well as their active role in supporting the patient. At the same time, such sessions would provide the necessary context for asking questions, clarifying uncertainties, and strengthening mutual trust between the patient, the family, and the healthcare team [50].

In the long term, the implementation of family-oriented guidelines would represent an essential tool for standardizing and harmonizing the information provided, ensuring access to scientifically validated knowledge. These guidelines could include detailed explanations about the structure of cardiac rehabilitation programs, the main clinical and paraclinical stages the patient undergoes, and concrete recommendations on how family members can contribute to treatment adherence, lifestyle modification, and sustained patient motivation. Furthermore, they could highlight practical strategies to ensure that family involvement does not create unnecessary pressure or conflicts, thereby preventing any negative impact on the patient–family relationship or on the emotional balance of both parties. Such an integrative approach would strengthen the family’s role as an active partner in the therapeutic process, contributing not only to better clinical outcomes but also to improved patient quality of life and to a more balanced and sustainable family dynamic.

## Figures and Tables

**Table 1:** General data of included articles.

Year, Author and Country	Name of the Article	Number of Participants	Sex and Age of Participants	Patient’s Diagnostic	Study Type
Vahedian-Azimi et al., 2024, Iran [13]	Cardiac Rehabilitation Using the Family-Centered Empowerment Model is Effective in Improving Long-term Mortality in Patients with Myocardial Infarction: A 10-year Follow-Up Randomized Clinical Trial	70 patients	61.40 ± 12.83 Years, 65.7% males.	Myocardial infarction	Randomized Clinical Trial
Zhang et al., 2024, China [14]	Clinical Effects of Hospital-Family Collaborative Cardiac Rehabilitation Training on Patients with Heart Failure after Cardiac Valve Prostheses	201 patients	Mean age 67 years, 61% males.	Heart failure	Retrospective study
Birtwistle et al., 2020, UK [15]	Family support for physical activity post-myocardial infarction: A qualitative study exploring the perceptions of cardiac rehabilitation practitioners	14 cardiac rehabilitation practitioners	No data available	Myocardial infarction	A qualitative study
Ma et al., 2024, China [16]	Needs and Constraints for Cardiac Rehabilitation Among Patients with Coronary Heart Disease Within a Community-Based Setting: A Study Based on Focus Group Interviews	11 patients	55–80 years, 72.72% females	Coronary heart disease	Semi-structured interview
Tuomisto et al., 2018, Finland [17]	Family Involvement in Rehabilitation: Coronary Artery Disease–patients’ perspectives	169 patients	61–74 years, 76% males	Coronary artery disease	Descriptive cross-sectional study
Hagan et al., 2007, Australia [18]	Financial, family, and social factors impacting on cardiac rehabilitation attendance	10 patients	43–82 years, 80% males	Myocardial infarction	Semi-structured interview
Astin et al., 2008, UK [12]	Family support and cardiac rehabilitation: A comparative study of the experiences of South Asian and White-European patients and their carers living in the United Kingdom	65 patients	40–83 years, 55.38% males	Angina (32%), Myocardial infarction (42%), CABG surgery	Semi-structured interviews
